# A Case of Cyanosis With Saturation Gap: Dapsone-Induced Methemoglobinemia

**DOI:** 10.7759/cureus.31684

**Published:** 2022-11-19

**Authors:** Mahammed Khan Suheb, Farheen Naaz, Tyler K Anderson, Alexander McClanahan

**Affiliations:** 1 Internal Medicine, University of Nebraska Medical Center, Omaha, USA; 2 Internal Medicine, Deccan College of Medical Sciences, Hyderabad, IND; 3 Orthopedics, University of Central Florida College of Medicine, Orlando, USA; 4 General Surgery, University of Central Florida College of Medicine, Orlando, USA

**Keywords:** dapsone-induced methemoglobinemia, auto-oxidation, saturation gap, methylene blue, cyanosis, dapsone side effect

## Abstract

Dapsone is an antibiotic used in the management of dermatologic infections and opportunistic infection prophylaxis in developed countries. Methemoglobinemia (MetHb) is a known complication of dapsone use that can result in cyanosis. MetHb is an aberrant form of hemoglobin produced physiologically by auto-oxidation. An impairment in the process of auto-oxidation due to genetic defects or the use of drugs/toxins causes its levels to rise. Management involves timely recognition and the use of methylene blue (MB) or ascorbic acid. We describe the diagnosis and management of a patient with acquired MetHb as a result of dapsone use.

## Introduction

Methemoglobinemia (MetHb) is a form of hemoglobin produced physiologically in which the heme iron is oxidized from the ferrous (Fe2+) state to the ferric (Fe3+) state. This Fe3+ state has a low oxygen-binding capacity leading to hypoxemia [1]. MetHb is a condition that can be severe or even fatal if left untreated [2]. There are two types of MetHb, hereditary and acquired [2,3]. Here, we present a case of an 82-year-old female with dapsone-induced (acquired) MetHb, whereupon a timely detection of her condition and treatment led to an improved outcome in the intensive care unit (ICU). We report this case study to highlight the importance of the early recognition and management of methemoglobinemia.

## Case presentation

An 82-year-old Caucasian female was admitted with complaints of shortness of breath (SOB) and high blood pressure (BP). She was placed on a nasal cannula for low oxygen saturation on pulse oximetry. A review of her history and medications was conducted. She was started on dapsone and prednisone for a diagnosis of bullous pemphigoid by her dermatologist recently. Following this, her family members noticed a decline in her mental status, with episodic disorientation and mood fluctuations. She visited a neurologist who diagnosed her with dementia after a brain magnetic resonance imaging (MRI) revealed atrophy.

Physical examination at the hospital revealed a blood pressure of 163/98 mmHg with a heart rate of 96 beats per minute (bpm) and oxygen saturation of 86% on 3 L of nasal cannula. She was awake with no signs of acute distress but had a pale appearance with cyanosis noted on nail beds, lips, and periorbital areas. Arterial blood gas on 2-3 L of nasal cannula showed a pH of 7.42, partial pressure of carbon dioxide (pCO2) of 34, and partial pressure of oxygen (pO2) of 84. This test was repeated on a non-rebreather face mask with a pH of 7.47, pCO2 of 26, and pO2 of 84.

The patient was administered 20 mg of labetalol to control her high BP. With declining mentation and worsening hypoxia despite oxygen supplementation, the patient was transferred to the ICU for close monitoring. A review of her symptoms, medication history, and arterial blood gases suggested a diagnosis of MetHb. MetHb levels were 39%. Other laboratory tests, including hemoglobin levels, were unremarkable. A chest X-ray did not show any infiltrates or consolidations. Dapsone was identified as the possible culprit for methemoglobinemia. The patient was started on nebulized bronchodilators and intravenous methylene blue (MB). MB was given intravenously at a 1 mg/kg dose over five minutes. Thereafter, her dyspnea and cyanosis improved remarkably over the next few hours. Also, methemoglobin levels and oxygenation improved remarkably with the early institution of methylene blue. The patient recovered well and was transferred out of the ICU three days later.

**Figure 1:**
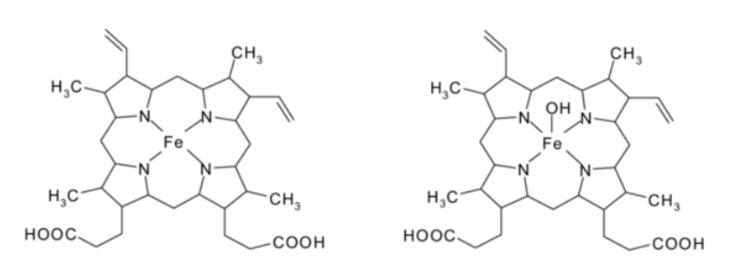
The difference between hemoglobin (left) and methemoglobin (right) is that the iron moiety of the heme groups is in the ferric/reduced form rather than the ferrous/oxidized form, respectively. Image reproduced from “Cataldo F: Ozone degradation of biological macromolecules: proteins, hemoglobin, RNA, and DNA. Ozone: Sci Eng 2006, 28:317-28” [4]

**Figure 2:**
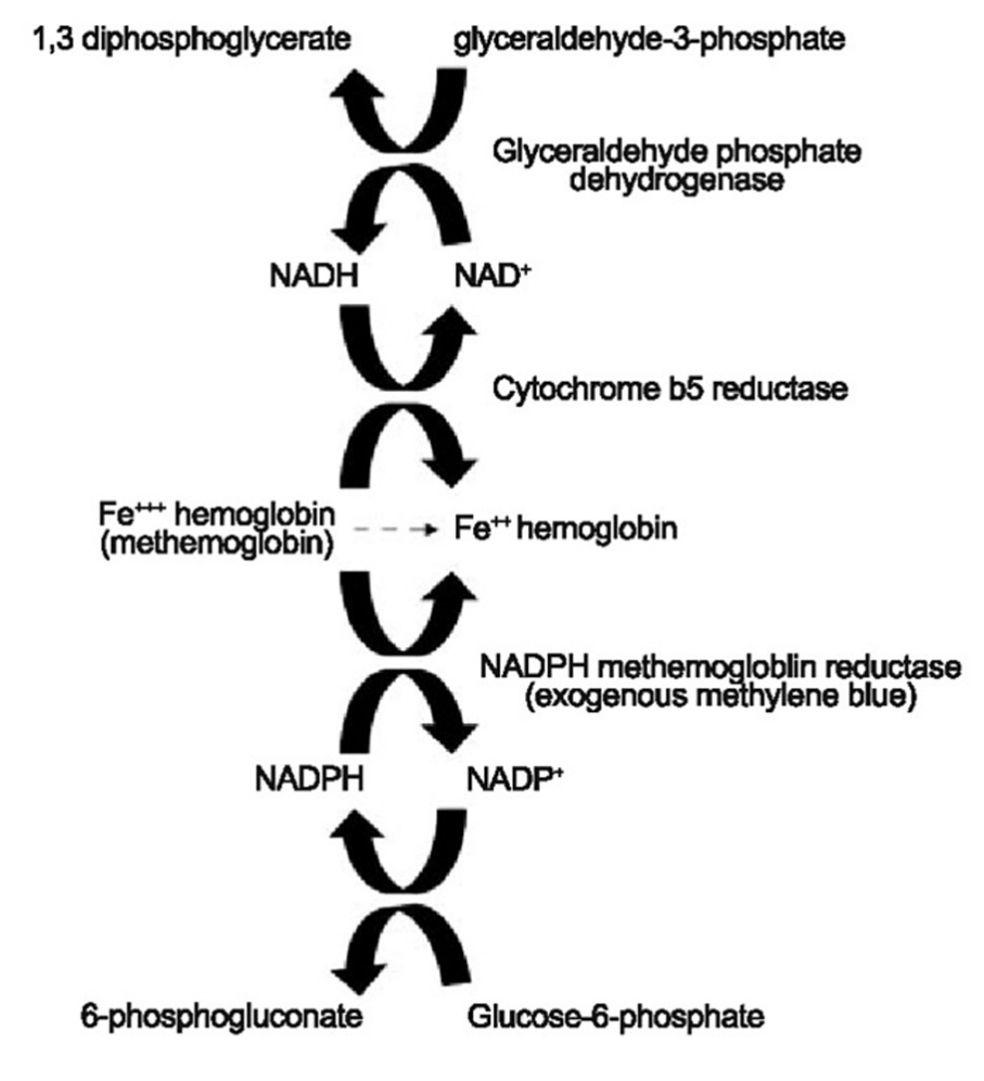
The natural mechanism of action for the reduction of MetHb (Hb-Fe+3) via cytochrome b5 reductase (Cb5R). Methylene blue bypasses this enzyme and reduces MetHb more rapidly, allowing for the resolution of symptoms. NAD^+^: nicotinamide adenine dinucleotide, NADH: reduced NAD^+^, NADP^+^: phosphorylated NAD^+^, NADPH: nicotinamide adenine dinucleotide phosphate Image reproduced from “Hayes D Jr, Roessler-Henderson KM, Davenport SM, Collins PB, Ballard HO: Dyspnea in a lung transplant recipient. Respir Care. 2010, 55(5):626-9. PMID: 20420735” [5]

Figure 1 shows the difference between hemoglobin and methemoglobin, and Figure 2 shows the natural mechanism of action for the reduction of methemoglobin.

## Discussion

Bullous pemphigoid results from the autoimmune destruction of epidermal cells leading to skin blistering [6]. It commonly affects the elderly population and is typically treated with steroids. However, sometimes, life-threatening adverse events can limit the use of prednisone. In such cases, steroid-sparing agents such as azathioprine and dapsone are commonly used as adjuvant therapy [7].

Dapsone is an anti-inflammatory and antiparasitic drug used worldwide to treat malaria, leprosy, and blistering skin diseases [8]. In the United States, it is mainly used to treat bullous dermatosis, pyoderma gangrenosum, and dermatitis herpetiformis [9]. Dapsone is metabolized by the liver and undergoes N-acetylation and N-hydroxylation, producing powerful oxidants contributing to its hematologic adverse effects [10]. Additionally, it undergoes enterohepatic recirculation and, as a result, has a long half-life (>30 hours) [11].

Dapsone is associated with potentially life-threatening adverse events, including MetHb, hypersensitivity syndrome, and dose-dependent hemolytic anemia [12]. According to recent studies, it is shown to be a leading cause of drug-induced methemoglobinemia [13]. Barclay et al., in their review, reported cases of dapsone-induced methemoglobinemia (DIM) between 1997 and 2011, suggesting that the incidence of DIM occurs even at therapeutic doses. The study concluded that timely intervention of severe cases is associated with lower mortality [2]. A list of medications and chemicals that may cause acquired MetHb is highlighted in Table 1. The diagnosis of methemoglobinemia is based on clinical manifestations and laboratory results. Arterial blood gases, pulse oximetry, and methemoglobin levels are used for diagnosis. A concentration of methemoglobin above 5% suggests methemoglobinemia [14]. Symptoms usually correlate with MetHb; however, levels may not correlate every time; therefore, a high index of suspicion is essential for diagnosing this condition [14,15]. In our patient, MetHb levels were at 39%. Given her symptoms of worsening mentation, refractory hypoxemia, and elevated methemoglobin levels, we decided to proceed with MB. The optimal dose for treating MetHb remains unclear; we followed evidence based on the literature review [16]. No second dosing of MB was needed as her cyanosis and MetHb levels improved with the first dose. MB functions by oxidation to leucomethylene blue, which acts as an artificial electron acceptor to MetHb, restoring it to hemoglobin (Figure 2) [12,16]. Another treatment option in the management of MetHb is ascorbic acid [17]. However, MB is preferred due to its rapid onset of action and dramatic improvement observed with treatment [13,17]. The use of MB is limited in glucose-6-phosphate dehydrogenase (G6PD) deficiency (hemolysis) and the use of serotonergic drugs (serotonin syndrome) [18,19]. In such situations, ascorbic acid can be used when MB is contraindicated or unavailable. Exchange transfusion and hyperbaric oxygen have also been reported to be beneficial in severe conditions [20].

**Table 1:** Common medications and chemicals known to cause methemoglobinemia.

Medications and chemicals
Chloroquine
Dapsone
Local anesthetics - benzocaine, prilocaine, and lidocaine
Metoclopramide
Rasburicase
Quinone
Sulfonamides
Food and beverages
Food products that use nitrates as preservatives
Mushrooms
Green leafy vegetables and root vegetables
Chemicals
Acetanilide (used in varnishes, rubber, and dyes)
Anilines and aniline dyes (e.g., diaper, laundry marking inks, leather dyes, and red wax crayons)
Naphthalene
Antifreeze
Nitrates and nitrites

## Conclusions

This case emphasizes the awareness of drugs likely to cause MetHb. In cases of cyanosis with a saturation gap not improving with oxygen, methemoglobinemia should be high on differential diagnosis. A good history, review of medications causing MetHb, and timely recognition of this rare condition can help decrease morbidity and mortality associated with dapsone-induced MetHb. Methylene blue is preferred over ascorbic acid due to its rapid onset of action and faster response.
